# Couples Coping With the Serious Illness of One of the Partners

**DOI:** 10.3389/fpsyg.2021.638938

**Published:** 2021-04-30

**Authors:** Hélène Riazuelo

**Affiliations:** ^1^UFR SPSE, UR 4430 CLIPSYD - A2P, Université Paris Nanterre, Nanterre, France; ^2^Nephrology Psychosomatic Unit, Aura Paris Plaisance (APP), Paris, France

**Keywords:** serious somatic disease, chronicity, spouse, partner, couple, reworking of family relations, transmission, filiation

## Abstract

Chronic kidney failure is a serious somatic disease. Addressing the issue of living with a chronic disease means fully considering the patients’ entourage, their families, and those close to them, especially their children and spouses.

**Objectives**: The present paper focuses on the couple’s psychological experience when one of them suffers from a chronic disease, in this instance kidney disease. In particular, how is the spouse affected by the treatment provided? The aim is not only to see how care for sick people can be improved, but also, more specifically, how relatives and especially partners can receive attention.

**Methodology**: A qualitative approach is not only adopted, being based on the psychotherapeutic follow-up of the partners of patients with chronic kidney disease, but also of the patients themselves, addressing the matter of their life as a couple. Three couples were considered, and two case studies are presented here. The issues that cut across these different situations are examined.

**Results and Discussion**: Some couples show considerable resourcefulness. However, over the years, that capacity for adaptation and inventiveness can also be interrupted by the periods of greater suffering and even despair, especially when the somatic pathology becomes chronic. Many spouses talk about how living with a sick partner weighs down on them, causing severe fatigue. Some aspects of the illness can also become traumatic. The disease regularly disrupts the daily life of the couple and the family. This leads to a reworking of family relations. Each couple has its own history with the condition. As it emerges, it can disrupt the bonds of filiation, especially when the illness is hereditary. Making psychological care more accessible to the partners involved constitutes a major challenge for our hospital care systems.

## Introduction

Major challenges currently exist in Europe and indeed worldwide to provide better management of patients suffering from chronic and/or noncommunicable diseases, whose numbers are constantly on the increase. “Noncommunicable diseases (NCDs), including heart disease, stroke, cancer, diabetes, and chronic lung disease, are collectively responsible for almost 70% of all deaths worldwide.”^1^ The available definitions focus mainly on the duration of the disease. According to the World Health Organization (WHO), chronic diseases are long-term conditions that generally progress slowly and require long-term treatment and care.^2^ However, definitions may vary from country to country and according to the underlying national objectives.

The proposed work aims to reflect on the couple’s psychological experience when one of them suffers from a chronic illness, in this instance kidney disease. People with chronic kidney disease and their partners will be more specifically considered. However, a significant part of our study is also relevant to couples with one partner suffering from another chronic disease. The pattern that emerges is one where those close to people suffering from such diseases still receive insufficient attention, with little or no support even when they feel the need for it. It is clearly essential to review practices in health services and to give consideration for the sick person as a whole, both somatically and psychologically, as also their family and friends.

Chronic kidney disease is a serious somatic disease characterized by impaired kidney function. As the kidneys no longer effectively filter the blood, the patient has to undergo dialysis or receive a kidney transplant. As treatment regimens improve, it is becoming increasingly common in the course of care for patients to go through both dialysis and transplantation. “We do not always realize it, as its work is so silent, but the kidney is an essential organ, and when it no longer functions, when we are, like me, in acute renal failure, all the other functions are distressed: if we do nothing, we die of poisoning” is how the novelist Nathalie Rheims so appositely described her condition (Rheims, 2019, p. 18).

People with the disease suffer from a chronic and potentially fatal illness. They can no longer “self-preserve” (Cupa, 2007). This is a “clinic of reality” (Raimbault, 1982). That one’s very survival depends on bringing a machine or someone else’s organ to intervene in the body is a staggering realization. A number of authors have stressed this (Becker et al., 1978; Carbonell, 1978; Cupa-Pérard, 1985; Cupa, 2000, 2002, 2009; Causeret, 2006; Riazuelo and Cupa, 2013; Riazuelo, 2016, 2020; Paumier-Bidault and Michel, 2018). The three weekly sessions of dialysis provide an incessant reminder of possible death.

The risks of depression are regularly highlighted in the international literature, both at a medical level and in the field of health psychology. There are numerous references in this field (some recent ones being Bonenkamp et al., 2020; Nair et al., 2020; Schouten et al., 2020 etc.). These studies seek to establish scales (of depression and anxiety in particular) and relevant and operative measures, particularly for preventive purposes, or to provide a comparative analysis of the treatments proposed (hemodialysis in the center or at home, peritoneal dialysis, before and after a transplant, etc.).

At a more psychodynamic level, Marty (1980) writes of “operative thinking” anchored in the factual. A lack of affect and a modification of the representational framework (in the sense of A. Green) can be observed as feelings and emotions no longer interconnect. J. MacDougall speaks of these somatic manifestations as “theaters of the body” as “crushing” these patients and “of their emotional state in the face of almost any situation capable of mobilizing emotionally charged performances. The curtains were somehow tightly closed on the psychic stage, no sound reached the ears of those outside, and yet a drama was played out on the inner stage of the one whose very life was threatened” (MacDougall, 1990, p. 15). They are regularly caught up in the present and enduring reality of the illness.

Medical progress has pushed back the limits of mortality. This is all about growing up, living, and aging while striving to survive a serious illness. The question of the entourage of sick people, their families, those close to them, children and spouses, then arises. The bonds with others are central to everyone’s lives. Maintaining them involves investing in one or more other people who invest in you in return. It is all about the friendly, loving, and family sphere that brings what is needed for a rich emotional life. This takes on its full meaning when the vicissitudes of life render one more vulnerable. The strength of such bonds can take on an essential and sometimes vital dimension (Bacqué, Baillet, 2009). It should be emphasized that the relationship with the medical and care teams is central to day-to-day care and treatment and, more generally, to the relationship between sick people and their illness.

The importance of the relational realm of the friendly and loving environment for patients emerges chiefly in research focused on the quality of life of sick people (Cupa, 2002; Morelon et al., 2005; Belasco et al., 2006; Draskic et al., 2011; Czyzewski et al., 2018; Schouten et al., 2020). Some works focusing on the relatives of patients with chronic kidney disease have also been published (Kaye et al., 1989; Devins et al., 1997; White and Grenyer, 1999; Gee et al., 2005; Belasco et al., 2006), and there has been a succession of further articles in recent years (Roques and Proia-Lelouey, 2015; Gilbertson et al., 2019; Jean-Dit-Pannel and Thomas, 2019 among others). These studies describe how the sick person not only finds support from their partner, contributing to their well-being, but also underline the draining effect this can have on that family member (Kaye et al., 1989). Conversely, some patients insist on the overbearing nature of the role their spouse takes in daily life and care, which can undermine the relationship within the couple (Devins et al., 1997). Multiple scenarios arise. In some instances, the partner becomes a kidney donor for his or her partner. Each couple has its own history more or less directly relating to dialysis or transplantation. Some patients and couples choose to “leave the illness behind in the hospital.” Dissension too may emerge. Despite the multiplicity of situations, all the authors consulted describe a situation that can be extremely distressing for relatives (White and Grenyer, 1999; Ekelund et al., 2004; Gilbertson et al., 2019).

The past few years have seen an emergent literature on “carers” and “family carers.” The term is being used increasingly, often to refer to those looking after people with a severe motor impairment, a severe psychiatric disability, Alzheimer’s (Schuster and Pellerin, 2019, for example) or cancer (Jarrossay and Paternostre, 2020, for example). These people come in on a regular basis to help in a non-occupational capacity with the activities and tasks of daily life. Their workload is gradually being recognized in many countries.

In the medical field, and more particularly in nephrology, Gilberston et al. identified 61 studies in 2019 that focused on the issue of family caregivers of dialysis patients. This figure is interesting in light of the fact that just 10 years ago; such research was still extremely limited. When carrying out this systematic review of the literature, they noted the difficulties involved in making comparisons given the extreme diversity of the methodologies adopted. However, they point out that they regularly identified a number of salient features. Firstly, the quality of life is worse among these carers than in the general population but still remains comparable to that of carers devoted to other chronic pathologies. Workloads are also heavier. This improves when the patient is transplanted. However, depression is less common among carers than among those suffering from kidney disease. Meanwhile, the cases of depression are regularly comparable or slightly higher for carers than for the general population. It should be noted that caregivers are most often married women.

These observations are interesting and help to contextualize our arguments. Above all, the concern is to clarify the experiences of family carers of people with chronic kidney disease by looking at what they can deliver and develop during psychotherapeutic follow-up. The aim is to adopt a psychodynamic approach to investigate some of the complex aspects of these situations and what lies at stake in the couple’s relationship.

## Methodology

The methodology adopted was qualitative. The clinical material derived from several psychotherapeutic follow-ups with a psychoanalytical orientation. These follow-ups were conducted in a nephrology department within the framework of the Nephrology Psychosomatic Unit.^3^ Follow-up programs were initiated for three partners (wives in the present instance) in response to their request for psychotherapeutic assistance. Their husbands or partners were dialysis patients at the center. The carers did not remain at the bedside during hospital treatment sessions although they participated actively in at-home dialysis. We met them alone and took detailed notes on what they reported as they talked about their relationships.

In each case, the support lasted at least 3 years. The sessions were regular, with one session per week or every fortnight for the third. Each situation was considered as a case study and cross-cutting themes were identified between them. For ethical reasons, all situations are made anonymous.

The following Table 1 gives a summary at start of psychotherapeutic follow-up:

**Table 1:** Some data regarding subjects we met.

Partner	Age	Living as a couple since	Medical follow-up time	Session frequency	Partner’s type of medical treatment	Partner’s dialysis duration
Nicole	65 years	42 years	5 years	Every week	Dialysis centre	6 years
Séverine	62 years	38 years	3 years 8 months	Every fortnight	Dialysis centre	15 years
Marie	58 years	27 years	3 years	Every week	Dialysis centre	2 years
**Patient**	**Age**	**Living as a couple since**	**Medical follow-up time**	**Session frequency**	**Type of medical treatment**	**Dialysis duration**
Paul	75 years	33 years	3 years 4 months	Every fortnight	Dialysis centre	10 years and a half
Alexandre	52 years	8 years	3 years 6 months	Every week	Dialysis centre	30 years
Louis	55 years	30 years	4 years 3 months	Every week	Dialysis centre	7 years

Attention here will first be devoted at greater length to two psychotherapeutic follow-ups. The first case was that of a woman whose husband had been suffering from chronic kidney disease for several years while the second concerned a dialysis patient who regularly talked about his couple during interviews. These two individuals’ narratives provide a valuable insight to help better understand their individual experiences in that clinical situation, giving the complementary perspectives of the spouse on the one hand and the sick person on the other.

## Clinical Results

### “Time Goes by, and Who Am I? Just Another Carer”

Nicole requests a psychological consultation when her husband has already been on dialysis for many years. She arrives very tired and depressed. She says she knew about her husband’s kidney problems from the early days of their married life. Gradually the disease took hold and dialysis became inevitable. She had been alongside him from the start, supporting him at each stage, managing the medication, organizing appointments, and resolving travel issues. She also oversaw a large share of their daily life. She relied heavily on assuming her role as a grandmother, relishing her grandchildren’s laughter echoing around the house. There was no real respite, but at least the children’s presence boosted her.

Her reasons for asking to meet a psychologist were largely due to her feeling unable to imagine her future without being overwhelmed by anxiety. She felt that even the slightest mishap might lead to total disruption. She became obsessed with this fear. The trepidation relating to the illness meant she no longer dared make plans for travel or home improvement work. Even suggesting that they take a weekend off together from time to time proved impossible for fear of being turned down by her husband. This led to her just living despondently from day to day. Her discourse over the months revolved around the drudgery of their daily life with nothing positive to add except to say that she knew her husband did all he could, that the disease had “struck him” and that he was in no way responsible. Her own good health became a source of guilt and she often had to rein in her own aggressiveness, finding it difficult to put words to her pain, all the more in so far as she regularly had the feeling that she had been reduced to simply being the servitor to a sick person. Nicole felt less and less listened to: “At least we take care of him, we take his pulse, we listen to his heart… But nobody’s there for me. I do not even have the right to take a break.” She told me, for example, that she had recently accompanied her husband to a consultation with a specialist and that when she entered the surgery the doctor welcomed them in and pointed to a chair saying “Here’s the seat for the carer.” That short phrase had echoed back to her over and over, summing up the level to which she had been reduced. She was no longer really “Nicole,” “Mrs T.” or even “the wife of” but merely the helper of a sick partner. Then, indulging in a swing of mood, she explained to me that she did not consider herself as a caregiver. She could not bring herself to do so. Knowing all the doses of medications by heart, taking his blood pressure, managing certain dressings, and so on she was able to find a place alongside her husband, almost forgetting her role before the disease invaded their lives. She reflected that she had just become one of her husband’s caregivers. This was sometimes tinged with a hint of rivalry. The impression of being in control of the disease gave her the impression that she was somehow mastering the situation in the face of certain anxieties, especially relating to death.

Over time, she found that the disease invaded what bound her to her husband. There was never a break and there would never be a cure as such. The treatment allowed her husband to survive and would always be necessary. She felt caught up in this helping relationship. She came round to thinking, on some days, that she was sacrificing herself and would never be able to get out of that relationship that she described as a “stranglehold.” How could her life be summed up that way? She also stressed that being just a caregiver also meant reducing her husband to being a sick person only. She realized that she was adopting a relationship of overprotection which, combined with the absence of any sex life and the decline of her sick partner, was almost reminiscent of the bond between mother and child. She added that with the disease she no longer recognized her husband’s body. She sometimes feared “damaging” that body already weakened by illness. That illness was omnipresent in their intimacy. Sexual relations had become increasingly rare, frustrating and then non-existent. From the onset of the disease, their sex life had been undermined, as has been observed for so many couples (cf. Ekelund et al., 2004). Nicole not only talked about the waning of desire but also about the fear, sometimes as a form of reaction, of further harming his sick body. She remained frightened after her hand accidentally brushed the fistula and she distinctly perceived his pulse throbbing on the surface of his skin. She struggled not to reduce her husband to that sick body. Sometimes she found herself fixated on certain scars, remembering how he had found the will to fight back against the disease. That heroic dimension enabled her, then, to continue investing in that body, in the hero facing off the disease.

Suppressing her emotions so as better to support her husband as she thought, she gradually isolated herself in her distress. She slowly became depressed, withdrew socially, without being able to talk about her fears, her anxieties and what made her aggressive. She had moments of anger that helped her to fight off depression but that also made her feel guilty, further depressing her. She felt caught up in a vicious circle. The first consultations were essentially devoted to letting that pent up frustration find expression. Gradually, she came round to talking about herself, telling her story. In a game of disinvestment and reinvestment, she was able to resume certain leisure activities, see her husband a little differently, even though any somatic problem that arose would open the way for diffuse anxieties to affect them both, each of them mirroring the other.

### “Everything Against the Other”

Paul was a 75-year-old patient who had been on dialysis for just over 10 years. Psychiatric consultations had lasted a little more than 3 years with one session every fortnight. His wife had not received any special attention from the Dialysis Unit but had been very much present alongside her husband. She was seen to accompany her husband for each of his treatments at the Unit. She prepared his dialysis bag for him and provided him with a book, food and a blanket. She would leave after his being connected to the machine and having received a text message from him telling her that everything was fine, coming back 4 h later to pick him up. Sometimes she stayed in the waiting room throughout dialysis. During those periods of separation, they phoned each other or sent text messages.

During our consultations, they would sit next to each other. As I moved over to greet them, they both stood up together. She helped him a little while he leaned on her and in turn helped her to her feet. I sometimes had the feeling that she was somehow entrusting her husband to me. She settled down to wait for him, filling in time by doing crossword puzzles. He took a long time before letting her go from him, even for the short time of a simple consultation. When separating, they were attentive to each other and rushing them was clearly not an option. Our conversations led to Paul telling me a lot about his wife. He first met her quite late in life at a business meeting outside his company. He had been sharing everything in life with her for more than 35 years. They had gone through the announcement of the disease together. Dialysis after dialysis, she was the one who placed the anesthetic patch on his fistula, for example (arteriovenous access for dialysis), to reduce the pain on the needle site. In the course of the interviews, Paul stressed how meeting his wife, willing and able to protect and care for him, was so vitally important to him. He explained to me that he had previously lacked real attention and “true care.” He added that he too was very much present alongside his wife. Their relationship was fusional.

After more than a year of consultations, he brought up the matter of separation in the waiting room. At first, he smiled at the idea, depicting that eventuality as a moment in suspension. He talked about it gently and tenderly, insisting on the solidity of their couple, the attentiveness they afforded each other. At times, a sense of unease laden with anxiety became apparent as he explained how it was important to prepare for the moment when they would have to take leave of each other for good. Discussing the matter of the disease, the guilt he felt for putting his wife through the trials of his illness and the likelihood of a fatal outcome were central to our discussions. He told himself that 1 day he would have to pluck up the courage to talk to her about his worsening state of health and the inevitable fatal outcome. They both followed changes in his condition together, reading the medical reports together, but never talking about it. “The matter of the illness and the background of my death became taboo,” he told me that “too painful even to contemplate.” During consultations that moment in the waiting room when they let go of each other’s hands with great delicacy to take leave of each other, and in a way put the issue of separation to work, came up regularly. Paul also made a connection with the care given him by his godmother when, as a child, he arrived at her home weak and undernourished and how during a walk to strengthen his muscles, he was able to let go of her hand and make his own way proudly and confidently. He also confided that he had been talking about his fears with his son and how he was counting on him to take care of his mother should anything happen to him.

## Discussion: The Couple Coping with Chronic Illness

These two series of consultations shed light on the complexity of what plays out within a couple when one of them is seriously ill.

In a cross-cutting manner (concerning six follow-ups, three partners and three people suffering from nephropathy), let us emphasize that a number of couples were seen to have demonstrated a considerable capacity for adaptation and inventiveness over the years. Some periods were clearly more painful and a source of suffering, especially when the somatic pathology worsened. The loneliness experienced by some partners was regularly expressed. While early on they said they felt supported, they found themselves being given less and less attention as time went by.

Furthermore, as outlined in the methodology, it was noted that over more than 20 years of clinical practice in a nephrology department and having followed several relatives of sick people, those coming in for psychotherapeutic care consultation were mainly wives. The literature too reveals that family carers are regularly wives. Gilbertson et al. (2019), who carried out a broad review of the literature on the situation of carers of people undergoing dialysis, found 61 studies on this issue, where the group identified were wives of patients (72.3% of hemodialysis patients and 20.6% of peritoneal dialysis patients).

The way the disease is managed can also vary. Many of the patients’ partners were heavily involved in the local community. This also provides another way of understanding the disease and feeling active. However, even though I only met female partners, it did not seem appropriate to confine my comments to them alone. Certain lines of thought and experiences are cross-cutting, shared, regardless of gender, when faced with a serious and chronic illness. The following are the main points.

### Guilty of Being in Good Health, Guilty of Being Sick: in Silence

Sometimes feeling guilty for being in good health, partners can enter into a sort of spiral, where they come to accept all the patient’s demands. In such moments, emotional distress can arise. They refrain from speaking out, avoiding even minor clashes that could mildly hurt the feelings of the sick partner. This was especially the case for Nicole. She wanted to be absolutely perfect, attentive to every detail, throwing herself into everyday tasks. She was the “good mother” who cared whatever she might be going through. There was no question of any form of self-indulgence, no other options were available to her as she kept telling herself, paradoxically sinking into passivity in her relationship with her husband, never answering back or refusing him anything. But there were times when she could hardly bear the state of dependency in which her husband regularly found himself. After a few months, she realized that she could also maintain this dependency herself. Her husband may regularly be exhausted by the illness, but gradually they would find common ground on what could be managed by one or other of them.

As she devoted herself to this care work, she managed to explain to me that while never overtly expressing her feelings, especially her ambivalence and aggressiveness, these could find indirect expression when dispensing care. For example, she sometimes went through the motions too fast, ripping off the armband of the blood pressure device, thus hurting her husband. The unconscious desire to assault her partner, urged on by death wishes, can overwhelm the spouse who then feels a painful sense of guilt. A guilt that can resonate. Paul too, for example, felt guilty toward his wife for being a sick husband.

Some partners regularly talked to us about their weariness, their sense of guilt at being exhausted and being unable to bear the place the illness occupies.

### Containing Diffuse Anxieties

In *Les Reins et les Cœurs*, N. Rheims narrates her experience. The narratives of this French writer provide, particularly, enlightening insights. She is currently undergoing a transplant as a result of kidney disease and her mother and grandmother also suffered from the same condition. “I can hear the regular noise of the machine filtering me like a sponge. I follow the path my blood takes. It leaves me, passes through the artificial kidney, then comes back, through the same orifice, into my veins, cleaned, washed out. Impossible to imagine this return journey between the inside and outside of the body without having gone through it. You also have to get rid of all that water that makes me a monster of the depths, something you hardly think about either. Dying submerged by your own fluids is like returning to the fetal stage, floating in your mother’s womb, but to drown there” (Rheims, 2019, pp. 77–78). The author captures the complexity of the treatment and how it chronically stretches the boundaries of within and without.

A similar picture emerged from the interviews. The difficulty of containing one’s anxieties, those that remain diffused in the space of the couple and the family, were mentioned repeatedly. The inevitable transformation imposed by dialysis can be experienced as a catastrophic change in the sense of W.R. Bion, where the patient feels powerless and overwhelmed. Commencing dialysis requires an intrapsychic and intersubjective reorganization. This means looking at oneself and one’s life without overly denying the loss of oneself. Faced with this complex situation, patients will use more or less onerous and effective strategies depending on their personality. At times when somatic disorders intensify, when complications suddenly arise or when pain builds up, the partner is largely drawn in to take on some of the burden, helping to contain the effects alongside the sadness, the fears and anxieties. The recurrent themes evoked related to anxieties of being drained (fear of the extra-corporeal circuit and that the inside will flow out) and those relating to death. These are often the most psychologically disturbing. That suffering can become unbearable when fear of dying becomes daily, coming in to undermine and subvert the continuity of daily life. The state of passivity, or even passivation, the dialysis patient is forced into can lead to dangerous forms of regression and pathological mourning. With the supportive partner too, there is a need to take an interest in the most basic forms of distress, to listen to the most primitive anxieties. They may be a depository or be traversed by formal signifiers (Anzieu, 1987) and archaic anxieties. The partner may be confronted with a sick person whose somatopsychic limits are threatened. They are, as it were, dislocated, pierced by the repeated drawing off of the blood, which then circulates outside the body. The phantasm of a “flayed body” emerged several times in interviews with Nicole.

### Trauma Transmission

There is also the matter of mental paralysis and traumatic metaphoric “break-ins” in the couple and/or the family. One author, E. Reinhardt, recounts his personal experience and the illness of his wife, suffering from cancer, in one of his narratives. The somatic condition is different but similarities can be drawn, especially in terms of the families’ experiences. He writes: “I told myself that the worst was not so much the illness, which was now being treated by the doctors, but the fear, the anxiety, the devastating panic. I was afraid she would give in to her illness” (Reinhardt, 2017, p. 12). Nicole evoked the same pervasive anxiety that her husband would let go and no longer fight back against the disease. A fear and distress she sometimes recounted repeatedly in a traumatic manner.

Nicole also recounted how she sometimes found her husband in such a state of distress that she was unable to find sufficient resources to calm him down. She ended up putting cool refreshing cloths on his face or simply holding his hand and talking to him gently. After that, she would take a while to calm down herself, repressing her inner turmoil for hours on end. Listening to Nicole, the notion of primary distress (*désaide*) came to mind, a prototype of traumatic situations, resulting from the inability to satisfy one’s needs without the help of the object (Laplanche and Pontalis, 1967).^4^ There is not only the idea of the subject’s inability to help themselves but also the lack of internal resources to deal with what they are experiencing. The dialysis patient may experience states of distress close to that of an infant unable to survive alone. It is commonly observed that when the disease has irreversibly taken hold and when death becomes imminent, those first infant relationships return to the forefront. “When death comes, the balances implicitly constructed and elaborated in what remains unspoken of the respective deficiencies and strengths will find themselves stripped, sometimes reinforced and sublimated, sometimes corroded and annihilated, but always disrupted. The exceptional and the unexpected disturb and upset, harass and erode the very roots of the relationship, in an ultimate explosion that everyone feels, though never willing to name it” (Ruszniewski, 1999, p. 89).

Containing anxieties, stemming the pain from time to time or on a daily basis is a daunting task that can weigh heavily on partners. This can be a strain on some couples, but others, like Paul and his wife, grew closer together. Multiple situations clearly arise.

Rearrangements within the family are significant and the children are particularly affected by this. The anxieties are pervasive, especially those relating to death, regularly breaking in on the couple or the family. The possibility of passing on the trauma is regularly there in the background. Relatives, spouses, and also children regularly conceal all that they themselves are going through, how they are challenged, sometimes made distraught, in order as they believe, to protect the sick person they love.

A number of kidney pathologies are hereditary (polycystosis, for example) and the matter of the disease arises in filiation, with the additional pain this entails. Returning to N. Rheims, she explains how the women in her family were all affected by the disease and unable to escape from it: “We had seen our mother on dialysis for 25 years. This was no illusion. She was receiving visitors at home, in her bed, hooked up to her machine. How could we forget this? How could we fail to imagine that it would affect us too? Our mother held the record for longevity in dialysis. I had also seen my grandmother, Alix, whom I adored, die of this terrible disease, and her sister Minka, who died a few months later, struck down by the same illness” (Rheims, 2019, p. 10).

There are so many different situations that cannot all be addressed here. The people we meet tell of the stories of couples who, despite the ordeals of the disease, stay together. Nicole sometimes brings up the possibility of a separation when some days turn out to be extremely heavy, but she regardless stays by her husband’s side. However, it should be remembered that some couples separate. Traumatic elements can weaken even the most united couples, break the family apart. The anxieties (in particular, the anxieties of death) are regularly pervasive and can upset, manhandle the closest relatives. It can also be a matter of patients who tell how they feel, find themselves progressively “damaged” by the disease and who say that they are gradually being withdrawn by their husband or spouse. However, it should be added that there are also couples younger than those mentioned above who refuse to have biological children so as to avoid taking the risk of passing on the disease. The illness emerges while they are still young and they make the often painful decision to make sure it ends with them. Others try and try again to have a child but the infertility caused by the pathology often leads to the couple remaining childless. The issue of fertility can be at the heart of couples’ concerns when the illness occurs at an age when they wish to become parents.

## Conclusion

In conclusion, one should bear in mind that renal disorders involve severe and constraining therapy. People with kidney disease have a special need to build on the relationships they have with others. This can hold in and contain the patient, maintain their somatopsychic limits that have become so uncertain, and remind them that those close to them hold them dear. This is part of a movement on the side of life.

This support provided by loving relationships helps to alleviate the deadly loneliness that the patient experiences at times. Partners play a major role in rehabilitation, care, and assistance for their sick loved one. Opening out to more psychological and psychotherapeutic consultations for partners and other family members is becoming essential in facilitating their access to psychological care. In consultation, there is a need to be attentive to the slightest movements from which the psyche emerges under the shadow of illness and death. Emotions gradually emerge, pockets of anger and sorrow which, when they do not unravel, can gradually link up in the space of psychotherapeutic work. The partners may also feel psychologically worn down by a reality that is just too burdensome. The work of hospital clinical psychologists is then to support the bond within the couple and be able to offer a supportive environment for the carers to work things out. If the place they occupy in daily life is based on guilt, a kind of assignment and obligation, this can tend toward a risk of collapse.

Lastly, let us conclude with a further quotation from N. Rheims, where she rebounds, finding renewed strength and turns again to life: “It is in the depths of this fatigue, unlike anything previously experienced, that I discover an unsuspected residual strength, that which makes us cleave to life, whatever the circumstances” (Rheims, 2019, p. 84).

## Data Availability Statement

The original contributions presented in the study are included in the article/supplementary material; further inquiries can be directed to the corresponding author.

## Ethics Statement

Ethical review and approval was not required for the study on human participants in accordance with the local legislation and institutional requirements. The patients/participants provided their written informed consent to participate in this study.

## Author Contributions

The author confirms being the sole contributor of this work and has approved it for publication.

### Conflict of Interest

The author declares that the research was conducted in the absence of any commercial or financial relationships that could be construed as a potential conflict of interest.

## Footnotes

^1^https://www.who.int/health-topics/noncommunicable-diseases#tab=tab_1

^2^idem.

^3^The Nephrology Psychosomatic Unit (AURA APP) is a team of four part-time clinical psychologists offering consultations for people with kidney disease. It also caters for the family and friends of patients followed in the unit, especially their partners.

^4^The term appears in his correspondence with Fliess, first in Manuscript G, 1895 and then in *Entwurf einer Psychologie* (Freud, 1895). It is used further in *Inhibitions, Symptoms and Anxiety* (Freud, 1926).
